# Dementia & Neuropsychologia: one year on

**DOI:** 10.1590/S1980-57642009DN20100001

**Published:** 2008

**Authors:** Letícia Lessa Mansur

**Affiliations:** Associate Editor

**Dementia & Neuropsychologia** has achieved its goal of publishing four issues in 2007, the year of its launch and now seeks indexation. A brief analysis of this course reveals numerous positive aspects which deserve mention.

1. National authors dealing with themes of a clinical nature, while also making room for more basic themes, have indicated the possibility of integrating these diverse areas of knowledge.
2. The plurality of methods employed in neuropsychology was explicit in case studies, studies using population samples and others of an epidemiologic nature. The pertinence of applying these methodological designs and their specificities has received recognition.
3. From a cognitive point of view, many aspects such as executive function, attention, gnosias, praxias, memory and language were addressed.
4. From a clinical point of view, themes related to various types of dementia, aphasias, dyslexias and frontal syndromes were dealt with as well as studies on the ageing process.
5. Studies on adults and the elderly including both healthy subjects and those with cognitive alterations were predominant, where these studies extended to include caregivers. The relevance of studies involving this age group is unquestionable. Research directed to health, throughout the world, have not been able to ignore the neurological disturbances frequent in the aged population nor have they offered quick and viable solutions for the supervened consequences.
6. Instruments for evaluation, such as tests and functional scales with translations and discussions about adaptation for the Brazilian culture were presented along with results from the local population samples.
7. Concern over evaluation was also permeated by the definition of diagnostic criteria and the terms used to designate syndromes and illnesses.
8. In the studies, Brazilian socio-cultural questions were very detailed and thoroughly investigated, with special emphasis given to schooling. In Brazil, in spite of the steady decrease in rates of illiteracy, we still face a vast contingent of functional illiterates. Furthermore, the educational profile outlined by the Brazilian Institute of Geography and Statistics (Instituto Brasileiro de Geografia e Estatística) indicates that the aged – a population having a high prevalence of neurological illnesses with cognitive alterations – have less schooling than the young.
9. In the articles we noted the use of cognitive evaluations that aim to obtain cultural norms of reference as well as norms for the creation of diagnostic criteria. Another interesting point was the use of cognitive evaluation in the continuum of the intervention process, to evaluate the functional evolution of the patients and the effects of intervention on various cognitive aspects. The exchange of experiments using diverse models and their results, are extremely important to direct and establish guidelines for care of patients, families and caregivers.
10. The multidisciplinary nature of the investigations is evidenced in most of the articles, demonstrating the advantages that integration brings for deepening and increasing knowledge.
11. The dissemination of congresses, symposia and other events in the area of neuropsychology, as well as the publishing of the summaries of these scientific meetings, serve as an opportunity to further motivate the exhibitor to formalize their studies into complete articles.
12. The desirable interchange among diverse Brazilian regions was another positive point and possibly a significant step toward stimulating Brazilian multi-centric studies. We are certain of having contributed to professionals and researchers, with specialized knowledge and similar efforts, bringing them closer together. This proximity is conducive for the exchange of experiences and access to the knowledge produced in other regions, constituting a bridge for theoretical and practical interchange in a country of contrasts, such as Brazil.

Neuropsychology is recognized as a new area and the exchange of information among in this field is essential and particularly necessary when considering rare or little-known diseases. In the case of dementias, for example, new roles in numerous areas of knowledge are being defined, not only involving differential diagnosis, but also evolution of the disease, development of individual and environmental interventions, as well as family counseling and staff education.

This positive outcome stimulates, for 2008, the optimistic expectation of consolidating the results achieved and increasing the number of readers and collaborators, both domestic and international.

**Letícia Lessa Mansur** Associate Editor

